# A Systematic Review and Meta‐Analysis of the Effectiveness and Cost of Single‐Site Robotic Surgery and Single‐Site Laparoscopic Surgery in Gynecological Diseases: The Era of Single‐Site Robotic Surgery May Have Arrived

**DOI:** 10.1002/cnr2.70327

**Published:** 2025-09-09

**Authors:** Jian‐Zhao Yin, Wei‐Feng Gao

**Affiliations:** ^1^ Jian‐Zhao Yin Department of Gynecology and Wei‐Feng Gao Department of Anesthesiology Gansu Provincial Hospital Lanzhou Gansu China

**Keywords:** gynecology, meta‐analysis, minimally invasive, single‐site Da Vinci robotic surgery, single‐site laparoscopic surgery, systematic review

## Abstract

**Background:**

The existing research data cannot fully prove the advantages of single‐site Da Vinci robotic surgery (RSS) compared with single‐site laparoscopic surgery (LESS) in the treatment of gynecological diseases.

**Aims:**

To evaluate the effectiveness and cost of RSS and LESS in the treatment of gynecological diseases. To provide a theoretical basis for RSS to replace LESS in the treatment of gynecological diseases.

**Methods and Results:**

A systematic search of PubMed, EMbase, and Wanfang (万方), three electronic databases for articles published up to December 31st, 2023, was performed by computer. After literature screening, data extraction, and quality evaluation according to inclusion and exclusion criteria, a meta‐analysis was performed using RevMan 5.1 software. A total of 16 articles were included, including 14 articles on effectiveness, with 2315 patients, and 2 articles on cost, with 544 patients. Compared with LESS, RSS was associated with a shorter total operative time for malignant tumor surgery (107 patients MD −24.01 min, 95% CI −32.40 to −15.62, *p* < 0.00001), less estimated blood loss (107 patients MD −53.60 mL, 95% CI −105.50 to −1.69, *p* = 0.04), and there was no significant difference in postoperative hospital days and postoperative complications between the two groups. The subgroup analysis of total operative time was carried out separately according to the time of study publication and different single‐point ports and robotic surgical systems. After 2020 and for using commercial single‐point ports other than the Da Vinci dedicated single‐point port, or using the fourth‐generation Da Vinci SP surgical system, there was no significant difference in total operative time between the two groups (1259 patients MD 16.91 min, 95% CI −9.38 to 43.19, *p* = 0.21) (354 patients MD 34.13 min, 95% CI −16.75 to 85.01, *p* = 0.19) and (645 patients MD 13.79 min, 95% CI −26.85 to 54.43, *p* = 0.51, respectively).

**Conclusion:**

The present meta‐analysis supports that, compared with LESS, in gynecological malignant tumor surgery, RSS takes shorter total operating time, less estimated blood loss, and has similar efficacy in postoperative hospital days and postoperative complications. However, the total hospital costs of RSS are higher than those of LESS. Therefore, if the cost of RSS can be reduced, RSS is a feasible surgical method for patients undergoing gynecological malignant tumor surgery. With the continuous updating of equipment and instruments and the widespread use of the fourth‐generation Da Vinci SP surgical system, the era of single‐site robotic surgery may be ushered in after 2020. However, due to certain limitations of this study, the above results must be interpreted with caution.

## 1 Introduction

Since the use of RSS in gynecologic surgery was approved in 2013 by the FDA [1], research on the feasibility and safety of RSS has never stopped. This is because single‐site surgery reduces adverse events such as bleeding from each trocar, wound infections, postoperative pain, hernia formation, intraabdominal organ injury, and multiple skin scarring caused by multiple piercings [2]. In addition, RSS overcomes the difficulties brought by LESS, such as the learning curve, instrument crowding, lack of triangulation, and loss of depth of perception or instability with current two‐dimensional flexible optics, and so forth [3]. It improves flexibility, three‐dimensional vision, and the comfort level of surgeons [4], so RSS has attracted increasing attention. However, it lacks tactile feedback. Therefore, there are more challenges than with the conventional laparoscopic systems when performing surgical tasks such as handling tissues like cardinal ligaments that are often stretched with tension. The field of view of the surgical assistant is different from that of the surgeon; the former sees a two‐dimensional field of view, while the latter sees a three‐dimensional field of view. This difference sometimes leads to discord between the doctors and slows down the procedure [1]. In addition, the high cost of acquisition, equipment, and maintenance of the Da Vinci surgical system is also the main disadvantage of the wider use of this technology. The cost is determined by the use of the surgical instrument, the duration of the operation, the cost of one‐time consumables, and the amortized capital cost per case of the Da Vinci surgical System [5]. Therefore, further instrument development is required to maximize the effectiveness of RSS and LESS [6], and further reduce the cost. However, the existing research data cannot fully prove the advantages of RSS compared with LESS. Therefore, this study focuses on the effectiveness and cost of RSS and LESS in the treatment of gynecological diseases through a systematic review. To provide a theoretical basis for whether RSS can replace LESS in the treatment of gynecological diseases, so as to guide clinical application.

## 2 Materials and Methods

### 2.1 Inclusion and Exclusion Criteria

#### 2.1.1 Study Type

All studies comparing the cost and effectiveness of single‐site Da Vinci robotic surgery versus single‐site laparoscopic surgery in gynecological diseases were blind or not, randomized or not, assigned to hide or not, regardless of study type. The language limits were Chinese and English.

#### 2.1.2 Study Subjects

All patients who needed single‐site Da Vinci robotic surgery or single‐site laparoscopic surgery were not limited by their race, nationality, disease type, course of disease, and surgical method. Exclusion: ① single‐site Da Vinci robotic surgery and single‐site laparoscopic surgery were used in the study of non‐gynecological diseases; ② studies unrelated to the effect and cost comparison of single‐site Da Vinci robotic surgery and single‐site laparoscopic surgery; ③ studies with unknown descriptions of study object, study method, and study results; ④ case reports, reviews, systematic reviews, meta‐analyses, and so forth.

#### 2.1.3 Intervention Measures

Single‐site Da Vinci robotic surgery in the experimental group and single‐site laparoscopic surgery in the control group. Other intervention measures were consistent between the two groups.

#### 2.1.4 Outcome Indicators

① Total operation time; ② estimated blood loss; ③ postoperative hospital days; ④ postoperative complications; ⑤ time for hysterectomy; ⑥ time for cuff closure; ⑦ mean serum hemoglobin drop; ⑧ total hospitalization cost.

### 2.2 Search Strategy

A systematic search of the PubMed (1881–2023), EMbase (1978–2023), and Wanfang (万方) database (1994–2023) to the comparative studies on the cost and effectiveness of single‐site Da Vinci robotic surgery and single‐site laparoscopic surgery in the treatment of gynecological diseases. The retrieval time was from the first issue to December 31, 2023. In addition, Google and other search engines are also used for supplementary retrieval, and references that have been included in the literature are tracked, and experts in the field are contacted to obtain information that cannot be found by the above retrieval strategies, so as to improve the recall rate of the literature. The following English keywords were used for the search: “robotic single‐site,” “robotic single‐port,” “robotic single‐incision,” “robotic laparoendoscopic single‐site,” “gynecology.” The following Chinese keywords were used for the search: “机器人单点手术” (robotic single‐site surgery), “机器人单孔手术” (robotic single‐port surgery), “机器人单切口手术” (robotic single‐incision surgery), “机器人腹腔镜单部位手术” (robotic laparoscopic single‐site surgery), and “妇科” (gynecology).

Take PubMed as an example, its specific search strategy (Box 1).

BOX 1 **PubMed search strategy.**

#1 robotic single‐site

#2 robotic single port

#3 robotic single‐incision

#4 robotic laparoendoscopic single‐site

#5 #1 OR #2 OR #3 OR #4

#6 Gynecology

#7 #5 AND #6

### 2.3 Literature Screening and Data Collection

The literature was screened independently by two authors according to inclusion and exclusion criteria, and cross‐checked: first, the title and abstract were read, duplicate or multiple submissions were excluded, and those that basically met the inclusion criteria were further read and included after meeting the inclusion and exclusion criteria. In case of disagreement, a third party was consulted to assist in making a judgment, and the authors were contacted to supplement the materials that were lacking as far as possible.

Data were extracted according to the established data extraction table. The extracted data content mainly includes: ① basic information of the included studies including the research title, the first author, the country, and time of publication; ② key elements of research design type and quality evaluation; ③ basic information of patients in the experimental group and control group including the number of included cases, age, BMI, uterine weight, previous history of abdominal surgery, history of pelvic adhesion, and histological type; ④ inclusion and exclusion criteria, surgical indications, surgical methods, the Da Vinci surgical platform, instruments and equipment used, and so forth for included studies; ⑤ total operation time, setup time, time for hysterectomy, time for cuff closure, docking time, estimated blood loss, mean serum hemoglobin drop, intraoperative complications, postoperative complications, postoperative hospital days, conversion rate, and total hospitalization cost.

### 2.4 Quality Assessment

The NOS (Newcastle‐Ottawa Scale) [7, 8] was used by two authors to evaluate the methodological quality of all the included studies. The content involved 8 items in 3 dimensions, and the total score was 9 points. The higher the score, the better the quality of the literature. The NOS scale was used because, except for one study in our meta‐analysis being a retrospective case–control study, all the other studies were retrospective cohort studies.

### 2.5 Statistical Analysis

Statistical meta‐analysis was performed under RevMan 5.1 software provided by the Cochrane Collaboration network. Mean difference (MD) and odds ratio (OR) were used for analysis. The confidence interval (CI) was set at 95%. The test level of meta‐analysis was *α* = 0.05. *χ* ^2^ test was used to test the heterogeneity of the results. When *p* > 0.10 and *I* ^2^ < 50%, the fixed effect model was used for meta‐analysis. Conversely, when *p* ≤ 0.10 and *I* ^2^ ≥ 50%, the random effects model was used for meta‐analysis. When there was significant clinical and statistical heterogeneity among the included studies, only descriptive analysis was used. When the mean and standard deviation were not provided, the equations proposed by Hozo et al. were used [9]. Because of the heterogeneity of the included studies, publication bias was not tested, and this is a confounding factor that may affect the methodological integrity of these tests.

### 2.6 Definitions

Total operation time (TOT): the time between the incision of the skin and the closure of the skin. Setup time (ST): the time required to set up the robot or laparoscope. Time for hysterectomy: the time from the removal of the uterus to the release of the uterus from the body. Time for cuff closure: the time from insertion of the needle holder into the abdomen to closure of the vaginal stump and removal. Docking time: the time from placement of the cannula until the last robotic arm locks the corresponding cannula. Postoperative hospital days (POD): the time from the day of surgery to the day of discharge. Estimated blood loss (EBL): calculated and determined by the difference between the volume of fluid used to irrigate and aspirate at the end of the procedure. Mean serum hemoglobin drop: the changes in hemoglobin values measured before surgery and on the first day after surgery. Intraoperative complications: any bladder, bowel, ureter, blood vessel, or nerve damage, or intraoperative blood loss greater than 500 mL. Postoperative complications: any adverse events that occur after surgery that meet the Clavien‐Dindo scale.

## 3 Results

### 3.1 Literature Search Results

**FIGURE 1:**
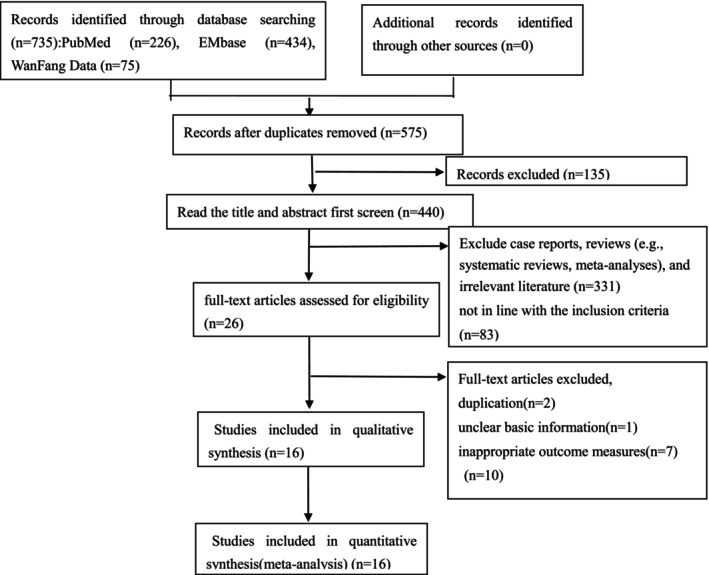
Search flow diagram.

According to the search strategy and data collection method, 735 articles were initially detected, and 626 articles were preliminarily excluded by excluding duplicate published studies and reading titles and abstracts. Then 85 articles were excluded by further screening by reading the full text and excluding articles that did not meet the inclusion criteria. Among these, 2 articles were excluded because they reported different sample sizes from the same study. Further, 8 articles were excluded by the main measurement indicators. Finally, a total of 16 studies were included [1, 4, 5, 6, 10, 11, 12, 13, 14, 15, 16, 17, 18, 19, 20, 21] including a total of 14 studies on effectiveness, with a total of 2315 patients. Among them, 725 patients received single‐site Da Vinci robotic surgery, and the remaining 1590 patients received single‐site laparoscopic surgery. Six of the studies performed total hysterectomy [10, 11, 12, 13, 14, 16], one supracervical hysterectomy [18], three adnexal surgery (removal of ovarian cysts or adnexectomy) [6, 15, 19], one radical hysterectomy with pelvic lymph node dissection for cervical cancer [1], one utero‐sacral ligament suspension with supracervical hysterectomy or total hysterectomy [4], and one study performed surgery for early‐stage endometrial cancer including a total hysterectomy, bilateral salpingo‐oophorectomy, and pelvic and/or para‐aortic lymph node dissection [17], and one study, some patients underwent total hysterectomy, some patients underwent ovarian cyst resection, and some patients underwent myomectomy [20]. While, A total of 2 studies on the total hospitalization cost, with a total of 544 patients. Among them, 50 patients received single‐site Da Vinci robotic surgery, 44 patients received single‐site laparoscopic surgery, and the remaining 450 patients received common laparoscopic surgery. All studies were published studies. Literature screening process and results (Figure 1). Basic characteristics of included studies and patients (Table 1). Basic characteristics of included surgeries (Table 2). Main results of included studies (Table 3).

**TABLE 1:** Characteristics of the included studies and patients (SS robotic vs. SS laparoscopic).

Author, year	BMI (kg/m^2^)	Weight of uterus (g)	Previous abdominopelvic surgery (%)	Pelvic adhesions (%)	Histological type (T/C) (%)					
	(T/C)	(T/C)	(T/C)	(T/C)	Uterine leiomyoma (%)	Adenomyosis (%)	CIN (%)	Endometrial hyperplasia (%)	Uterine prolapse (%)	Other (%)
Gardella 2023 [10]	23/26	58.8/79	N/A	N/A	16.39/38.46	N/A	1.64/0.64	26.23/41.66	N/A	55.74/19.23
Paek 2016 [11]	24.3 ± 2.5/24.0 ± 3.3	271 ± 119/249 ± 190	64/37.3	48/20.8	64/52	12/16.5	8/18.3	16/6.6	0/5.2	0/1.4
Lopez 2016 [12]	25.9 ± 6.1/28.8 ± 5.5	125.6 ± 68.5/117.9 ± 56.8	N/A	N/A	N/A	N/A	N/A	N/A	N/A	N/A
Akdemir 2015 [13]	28.5/27.45	192.5/117.5	75/70.6	N/A	N/A	N/A	N/A	N/A	N/A	N/A
Gungor 2017 [14]	28.7 ± 3.6/26.8 ± 3.6	N/A	30/24	N/A	N/A	N/A	N/A	N/A	N/A	N/A
Paek 2015 [15]	22.7 ± 2.9/23.0 ± 3.8	6.8 ± 2.6/8.9 ± 6.1 (tumor size) cm	30/37.7	65/40.8	N/A	N/A	N/A	N/A	N/A	N/A
Noh 2023 [16]	22.6/22.9	274.5/451.5	17.2/20	27.5/28.5	55.2/60	44.8/37.1	N/A	0/2.9	N/A	N/A
Sun 2021 [17]	25/24.5	N/A	31.8/33.3	N/A	N/A	N/A	N/A	N/A	N/A	N/A
Lee 2023 [18]	24.2 ± 4.06/24.69 ± 4.24	331.03 ± 199.61/380.40 ± 308.3	48.4/45.8	22.3/18.7	N/A	N/A	N/A	N/A	N/A	N/A
Kang 2023 [19]	21.4 ± 3.02/21.2 ± 2.92	7.9 ± 3.4/7.4 ± 2.5 (tumor size) cm	20.5/13.8	N/A	N/A	N/A	N/A	N/A	N/A	N/A
Moon 2018 [6]	23.1 ± 3.4/21.1 ± 3.0	5.23 ± 2.53/4.37 ± 2.14 (size of endometriosis) cm	N/A	N/A	N/A	N/A	N/A	N/A	N/A	N/A
Davila 2017 [4]	27/28	N/A	N/A	N/A	N/A	N/A	N/A	N/A	N/A	N/A
Gao 2021 [1]	23.76 ± 2.72/23.46 ± 2.28	2.21 ± 0.89/2.47 ± 0.87 (average diameter of cervical tumor) cm	N/A	N/A	N/A	N/A	N/A	N/A	N/A	N/A
Kim 2023 [20]	24.40 ± 3.74/24.64 ± 3.86 22.86 ± 4.39/22.07 ± 3.69 23.09 ± 4.19/23.92 ± 5.02	308.31 ± 231.03/362.94 ± 534.2	48.6/36.1 23.1/9.2 12.5/25	N/A	62.9/64.2	17.1/14.2	14.3/14.2	2.9/7.4	N/A	N/A
Woodall 2015 [21]	N/A	N/A	N/A	N/A	N/A	N/A	N/A	N/A	N/A	N/A
El Hachem 2016 [5]	26.3/27.9 (24.5/26.4 19.6/29.1 27.4/27.9)	107.2/225.0 (0/0 158.0/322.9 104.5/152.6)	N/A	N/A	N/A	N/A	N/A	N/A	N/A	N/A

Author, year	Country	Number of patient	Average age (years)	Interventions	Outcome indicators	Type of study
		(T/C)	(T/C)	(T/C)		
Gardella 2023 [10]	Italy	122/156	40.5/50.5	RSSH/LESSH	① ② ⑤ ⑦ ⑨ ⑩	Retrospective cohort study
Paek 2016 [11]	South Korea	25/442	48.0 ± 4.1/48.9 ± 8.7	RSSH/LESSH	① ③ ④ ⑤ ⑥ ⑦ ⑩	Retrospective cohort study
Lopez 2016 [12]	Canada	50/50	46.0 ± 9.4/45.4 ± 8.9	RSSH/LESSH	① ⑤ ⑦ ⑩	Retrospective cohort study
Akdemir 2015 [13]	Türkiye.	24/34	49.5/51.5	RSSH/LESSH	① ③ ④ ⑤ ⑦	Retrospective cohort study
Gungor 2017 [14]	Türkiye.	20/25	54.4 ± 7.8/52.4 ± 7.0	RSSH/LESSH	① ④ ⑤ ⑦	Retrospective cohort study
Paek 2015 [15]	South Korea	20/228	37.9 ± 10.5/41.3 ± 13.6	RSS/LESS	① ② ⑤ ⑥ ⑦ ⑩	Retrospective cohort study
Noh 2023 [16]	Korean	29/35	46.3 ± 5.0/45.9 ± 4.2	Mix RSSH/SPA‐TLH	① ② ③ ⑤ ⑥ ⑦ ⑨	Retrospective cohort study
Sun 2021 [17]	China	22/18	56/55.5	RSS/LESS	① ② ③ ⑤ ⑦	Retrospective cohort study
Lee 2023 [18]	South Korea	31/48	45.45 ± 3.89/46.69 ± 6.0	RSS/LESS	① ③ ④ ⑥ ⑦ ⑨ ⑩	Retrospective cohort study
Kang 2023 [19]	South Korea	78/87	33.5 ± 6.92/33.1 ± 7.09	RSS/LESS	① ⑤ ⑥ ⑦	Retrospective cohort study
Moon 2018 [6]	South Korea	68/52	32.4 ± 6.8/33.1 ± 7.9	RSS/LESS	① ⑤ ⑦ ⑩	Retrospective cohort study
Davila 2017 [4]	United States	5/13	70/68	RASS‐UTSLS/LESS‐UTSLS	① ④ ⑤ ⑦ ⑩	Retrospective cohort study
Gao 2021 [1]	China	32/35	51.63 ± 8.32/53.14 ± 8.14	RSS/LESS	① ⑤ ⑦ ⑨ ⑩	Retrospective cohort study
Kim 2023 [20]	South Korea	35/148 108/207 56/12	49.71 ± 7.86/50.89 ± 8.26 31.79 ± 10.31/28.78 ± 7.44 36.98 ± 7.14/41.42 ± 7.85	RSSH/LESSH RSSC/LESSC RSSM/LESSM	① ④ ⑥ ⑦ ⑩	Retrospective cohort study
Woodall 2015 [21]	United States	17/44/391	N/A	RSSH/LESSH/CL	① ⑧	Retrospective cohort study
El Hachem 2016 [5]	United States	33/59 (11/16, 1/17, 13/26)	56.2/54.2 (50.8/50.4, 43.0/48.4, 59.1/60.4)	RSSH/CL (RSSH/CL (Adnexectomy) RSSH/CL (supracervical hysterectomy) RSSH/CL (total hysterectomy))	① ② ⑤ ⑦ ⑧ ⑨ ⑩	Retrospective case–control study

*Note:* Outcome parameters: ① Total operation time; ② setting time; ③ time for cuff closure; ④ docking time; ⑤ estimated blood loss; ⑥ mean serum hemoglobin drop; ⑦ postoperative hospital days; ⑧ total hospitalization cost; ⑨ intraoperative complications; ⑩ postoperative complications; ⑪ time for hysterectomy.

Abbreviations: BMI, body mass index; T/C, experimental group/control group.

**TABLE 2:** Surgical characteristics of the included studies.

Author, year	Inclusion exclusion criteria	Indication and method for surgery	Methods for cuff closure	Da Vinci robotic surgical platform	Instruments and equipment	Other
Gardella 2023 [10]	Exclusion criteria: uterine size greater than 16 gestational weeks; a history of pelvic or abdominal radiation for a previous malignancy; any relevant disease that precluded a prolonged Trendelenburg position; severe hip diseases that precluded the dorso‐lithotomy position; and any cervical or endometrial cancer requiring a radical hysterectomy and/or lymphadenectomy.	Gynecologic benign disease; Total hysterectomy with or without bilateral salpingo‐oophorectomy.	The vaginal cuff was closed by a transvaginal approach for all procedures.	The Da Vinci Si platform (Intuitive Surgical, Sunnyvale, CA, USA).	The single‐site TM port, specific to the Da Vinci System SI was used.	Multi‐center study; Both groups were completed by one surgeon each.
Paek 2016 [11]	Patients who underwent RSSH or LESSH for benign gynecologic disease at Ajou University Hospital, regardless of the size of the uterus in all patients.	Gynecological benign diseases; Total hysterectomy.	In all cases, for the closure of vaginal cuff, we performed continuous running suture intracorporeally using a barbed suture.	The Da Vinci single‐site surgical platform (Intuitive Surgical Inc., CA, USA).	The Da Vinci Single‐Site Port.	Done by the same surgeon; Before inserting the RSS platform, we made a single multichannel port using a wound retractor and surgical glove and explored the pelvic cavity, while adhesiolysis was performed using laparoscopic instruments.
Lopez 2016 [12]	Included: ages 18–80; patients who underwent a hysterectomy for benign indications with or without salpingo‐oophorectomy; patients diagnosed with benign cervical cytology; and patients who were suitable candidates for laparoscopic surgery.	Gynecologic benign disease, total hysterectomy with or without salpingo‐oophorectomy.	The LESS group, an automated suturing device was used to close the vaginal cuff. The RSS group, the vaginal cuff was closed in a vertical fashion with non‐wristed instruments.	The Da Vinci Surgical System (Intuitive Surgical Inc., Sunnyvale, CA).	N/A	Multicenter study, done by three surgeons with similar surgical experience.
Akdemir 2015 [13]	Excluded: Confirmed or suspected gynecological malignancy; Uterus size > 16 gestational weeks; history of endometriosis, and co‐morbidities that are contraindications for laparoscopic surgery or prolonged Trendelenburg position, such as cardiopulmonary diseases.	Gynecologic benign disease, Total hysterectomy.	The RSS group, the vaginal cuff was closed intracorporeally with intra‐corporeal single stitches in all cases. The LESS group, the vaginal cuff was closed by single stiches and an extracorporeal knot‐tying technique.	The Da Vinci Single‐Site robotic surgery platform (Intuitive, Sunnyvale, Calif., USA).	The Da Vinci Single‐Site Port.	Done by the same surgeon.
Gungor 2017 [14]	Excluded: uterine size ≧ 16 gestational weeks; Morbid obesity (BMI ≧ 35 kg/m^2^); Active cardiopulmonary disease, or other risks for major surgery. Inclusion criteria for endometrial cancer: endometrioid type adeno cancer, grade 1 or 2 tumor, demonstrated < 50% myometrial infiltration which was confirmed preoperatively and intraoperatively, no obvious evidence of extra‐uterine spreading, lymph node and/or adnexal and/or cervical involvement at radiologic studies.	Gynecological benign diseases or early endometrial malignant tumors; All endometrial cancer patients underwent total hysterectomy and bilateral salpingo‐oophorectomy without pelvic and para‐aortic lymphadenectomy.	The RSS group, in 14 patients, vaginal cuff was closed robotically absorbable wound‐closure device, whereas vaginal cuff was closed vaginally in remaining 6 patients. The LESS group, an intracorporeal continuous suture vaginally in all patients.	The Da Vinci single‐site robotic surgery platform (Da Vinci Si Surgical System, Intuitive Surgical, Sunnyvale, CA, USA).	The Da Vinci Single‐Site Port.	Two centers, done by two doctors.
Paek 2015 [15]	It is identified 248 consecutive patients who underwent RSS or LESS surgery for adnexal tumors at Ajou University Hospital.	Adnexal tumor; adnexectomy or removal of ovarian cysts.	N/A	The Da Vinci Single‐Site Surgical Platform (Intuitive Surgical Inc., CA, USA).	The Da Vinci Single‐Site Port.	Done by the same surgeon; Before inserting the RSS platform, we made a single multichannel port using a wound retractor and surgical glove and explored the pelvic cavity, while adhesiolysis was performed using laparoscopic instruments.
Noh 2023 [16]	Excluded: Patients with cervical intraepithelial neoplasia, endometrial intraepithelial neoplasia, or suspected gynecologic malignancy.	Benign gynecological disease; Total hysterectomy.	The vaginal cuff was closed robotically in all patients with a continuous running suture using an intracorporeal barbed suture.	The Da Vinci Xi Surgical System (Intuitive Surgical, Sunnyvale, CA, USA).	A commercial single‐port platform, Glove Port (Nelis, Seoul, Republic of Korea).	Performed by the same surgeon, a hybrid RSS, first, the uterus was dissected by laparoscopy, and then the uterus was removed and the vaginal stump was sutured by Da Vinci robot.
Sun 2021 [17]	Inclusion: Preoperative imaging showed no evidence of metastasis to other organs; Uterine size < 12 gestational weeks; endometrial cancer stages IAto IB diagnosed by preoperative endometrial curettage or biopsy. Exclusion: supposed extensive adherences; large uteri requiring morcellation; And very morbidly obese women who could not sustain a steep Trendelenburg position.	Early‐stage endometrial cancer; Including a total hysterectomy, bilateral salpingo‐oophorectomy, and pelvic and/or para‐aorticlymph node dissection.	For closure of the vaginal cuff, we applied continuous running suture intracorporeally using a barbed suture.	Da Vinci Si surgical system.	Lagiport single port.	Done by the same surgical team.
Lee 2023 [18]	Excluded: Patients with other uterine diseases and total hysterectomy.	Uterine leiomyoma, supracervical hysterectomy.	The preserved cervix was sutured using barbed suture material.	The Da Vinci SP surgical system.	Da Vinci uses Uniport (UP04FSP‐A; Dalim, Seoul, Korea). Laparoscopically uses Uniport (UP03F) (Dalim, Seoul, Korea).	Done by the same surgeon.
Kang 2023 [19]	Inclusion: Patients who had not reached menopause, with or without extraovarian endometriosis including deep infiltrative endometriosis (DIE), underwent conservative surgery, and had AMH level test results before surgery, 2 weeks and 3 months postoperatively. Excluded: Those who had previously undergone ovarian surgery; those who underwent another gynecologic surgery (such as a hysterectomy or myomectomy) simultaneously.	Endometriosis; removal of ovarian cysts.	N/A	N/A	Use a single Port (Glove Port; Nelis Inc., Seoul, Republic of Korea).	Done by the same surgeon.
Moon 2018 [6]	Patients with endometriosis undergoing consecutive RSS or LESS surgery at Ewha Womans University Medical Center in Seoul, South Korea.	Advanced endometriosis; ovarian cystectomy and adhesiolysis.	N/A	Da Vinci Si system	The single‐site port, specific to the Da Vinci System SI was used. Laparoscopy uses GELPASS one‐port System (MEDEN Inc.).	Done by three surgeons with similar surgical experience.
Davila 2017 [4]	Inclusion: Patients who were at least 21 years of age with vaginal apex prolapse at pelvic organ prolapse quantitative stages 2–4 and who desired minimally invasive surgery. Exclusion: Not candidates for general anesthesia, previous history of vagino‐sacropexy, suspicious adnexal masses, history of pelvic inflammatory disease, morbid obesity (BMI ≧ 40), and prior or concurrent surgical history of rectal prolapsis.	For symptomatic vaginal apical prolapse; RASS: supracervical hysterectomy with uterosacral ligament suspension, LESS: total hysterectomy with uterosacral ligament suspension.	RASS‐UTSLS: performed with continuous running suture intracorporeally and tie a knot. LESS‐UTSLS: performed with continuous running suture through the vagina and tie a knot.	Da Vinci Single‐Site Surgery (Intuitive Surgical, Sunnyvale, CA).	Da Vinci uses the intuitive access and Gel‐POINT mini. Laparoscopic uses Gel‐POINT (Applied Medical, Rancho Santa Margarita, CA).	Done in two hospitals by two surgeons with similar surgical experience, applying a three‐point technique with gain excellent top‐end support.
Gao 2021 [1]	Exclusion: When patients with BMI > 28 kg/m^2^, or patients with tumor size > 4 cm, are considered unsuitable for use of these two methods.	Stage IB1 cervical squamous cancer; radical hysterectomy with pelvic lymph node dissection.	The vaginal cuff was closed by transvaginal running locking sutures.	Da Vinci Si Platform.	All use the LAGIS single‐site Port (LAGIS Enterprise Co. Ltd., Taiwan). The RSS group also used flexible robotic single‐hole instruments.	Done by the same surgeon.
Kim 2023 [20]	Patients undergoing RSS or LESS surgery at the Korea University Anam Hospital in South Korea.	Total hysterectomy, ovarian cystectomy, myomectomy.	N/A	Da Vinci SP Surgical System (Intuitive Surgical Inc., Sunnyvale, CA, USA).	Da Vinci uses different SP entry systems, including the Lapsingle Vision SP (SEJONG Medical Co., Paju, Korea), UNI‐PORT SP (DALIM Medical Co., Bucheon, Korea), and Glove Port SP (NELIS Co., Bucheon, Korea). Laparoscopy also uses different SP entry systems, including LAPSINGLE (SEJONG Medical Co., Paju, Korea), ONE‐PORT plus (MEDFINE CO., Hanam, Korea), and Glove Port (NELIS CO., Bucheon, Korea).	Done by six gynecological surgeons, surgical proficiency and surgical experience may vary.
Woodall 2015 [21]	Patients undergoing hysterectomy for any indication, with no staging or conversion to open.	Total hysterectomy.	N/A	N/A	N/A	Done by two surgeons with similar surgical experience.
El Hachem 2016 [5]	Inclusion: All female patients with a benign or malignant gynecologic condition undergoing CL or RSS hysterectomy or an adnexal‐only procedure.	Adnexal surgery: Included unilateral or bilateral salpingo‐only oophorectomy and ovarian cystectomy. Hysterectomy: includes total hysterectomy with or without adnexectomy, with or without retroperitoneal pelvic lymph node dissection.	N/A	Da Vinci Si Robotic Single‐site surgical platform (Intuitive Surgical, Sunnyvale, CA).	N/A	Done by the same surgeon; One case of IC stage ovarian malignancies in the RSS group underwent robotic bilateral salpingo‐oophorectomy, which was later converted to laparoscopic specimen extraction and greater omental excision.

**TABLE 3:** Main outcomes (SS robotic vs. SS laparoscopic).

Author, year	Total surgical time (min)	Setup time (min)	Time for hysterectomy (min)	Time for cuff closure (min)	Docking time (min)	Estimated blood loss (mL)	Mean serum hemoglobin drop (g/dL)	Intraoperative complications (%)	Postoperative complications (%)	Postoperative hospital days (days)	Conversion rate (%)	Total hospitalization costs (US $)
	(T/C)	(T/C)	(T/C)	(T/C)	(T/C)	(T/C)	(T/C)	(T/C)	(T/C)	(T/C)	(T/C)	(T/C)
Gardella 2023 [10]	165/120 (159.72 ± 48.76/122.89 ± 21.13)	15/10	—	—	—	93.52 ± 27.84/42.85 ± 27.37	—	3.28/1.92	2.46 (3/122)/5.12 (8/156)	3.5/2 (3.5 ± 0.75/2.35 ± 0.74)	5.74/0.64	—
Paek 2016 [11]	170.9 ± 65.5/88.3 ± 38.4	—	81.8 ± 49.6/52.2 ± 27.7	17.8 ± 10.8/15.0 ± 3.3	14.0 ± 4.7/0	20 (30)/50 (30)	1.6 ± 1.0/2.0 ± 0.9	—	0/2.49 (11/442)	3.5 ± 0.7/3.8 ± 1.4	No transfer to multi‐port or to open surgery	—
Lopez 2016 [12]	139.3 ± 45.8/121.0 ± 31.7	—	—	—	—	37.2 ± 30.7/42.0 ± 37.9	—	—	2 (1/50)/4 (2/50)	23.3 ± 9.1/31.9 ± 14.8 (0.97 ± 0.37/1.32 ± 0.61)	16/10	—
Akdemir 2015 [13]	98.5/86 (106.18 ± 28.76/88.98 ± 19.36)	—	53.5/43 (61.18 ± 19.51/45.87 ± 13.86)	21/26.5 (23.02 ± 6.42/28.04 ± 7.41)	5.5/0	22.5/25 (36.5 ± 5.25/40.25 ± 5.75)	—	—	0/0	1.6/1.8 (1.70 ± 0.51/1.95 ± 0.71)	No transfer to multi‐port or to open surgery	—
Gungor 2017 [14]	90/90 (98.17 ± 25.42/100.53 ± 35.63)	—	57.5/60 (64.18 ± 21.41/71.19 ± 29.27)	—	8.2 ± 1.7/0	40/50 (60.80 ± 48.17/72.39 ± 58.54)	—	—	0/0	1/1 (1.14 ± 0.26/1.26 ± 0.50)	No transfer to multi‐port or to open surgery	—
Paek 2015 [15]	91.1 ± 31.4/68.7 ± 34.0	8.1 ± 5.6/6.0 ± 2.2	57.4 ± 22.8/47.0 ± 31.0 (tumor resection time)	14.1 ± 1.3/12.4 ± 2.4 (incision closing time)	—	20 (40)/20 (40)	1.7 ± 0.6/1.7 ± 0.9	—	0/3.5 (8/228)	2.3 ± 0.6/2.7 ± 1.2	0/3.5	—
Noh 2023 [16]	103.0 ± 37.0/89.0 ± 43.0	6/5	52/52 (51.15 ± 4.19/51.45 ± 3.8)	15/10 (15.12 ± 2.22/10.27 ± 1.30)	—	150/100 (147.57 ± 29.61/110.87 ± 23.77)	1.4/1.7 (1.42 ± 0.24/1.74 ± 0.42)	6.9 (2/29)/2.9 (1/35)	0/0	2.2/2.4 (2.27 ± 0.24/2.53 ± 0.47)	No transfer to multi‐port or to open surgery	—
Sun 2021 [17]	95/125 (111.14 ± 51.51/123.19 ± 44.24)	8/4	—	21/30 (22.07 ± 11.88/30 ± 16.8)	—	50/85 (60.76 ± 55.47/85 ± 56.31)	—	—	0/0	2/3 (2.35 ± 2.37/3 ± 3.21)	No transfer to multi‐port or to open surgery	—
Lee 2023 [18]	111.26 ± 31.6/76.38 ± 29.27	—	62.16 ± 26.17/37.58 ± 12.65	11.71 ± 4.19/8.42 ± 2.59	3.59 ± 1.64/0	—	1.03 ± 1.51/1.22 ± 0.78	3.2 (1/31)/2.1 (1/48)	3.2 (1/31)/0	3.94 ± 0.68/3.71 ± 1.07	No transfer to multi‐port or to open surgery	—
Kang 2023 [19]	167.9 ± 63.5/93.7 ± 27.5	—	—	—	—	100/100 (100 ± 75.53/91.19 ± 56.53)	1.9 ± 1.0/1.7 ± 1.0	—	0/0	2/2 (2.35 ± 0.75/2 ± 0.1)	No transfer to multi‐port or to open surgery	—
Moon 2018 [6]	107.8 ± 37.6/76.9 ± 46.4	—	—	—	—	135.6 ± 143.9/57.1 ± 44.9	—	—	1.96 (1/68)/0	4.59 ± 0.58/4.58 ± 0.61	No transfer to multi‐port or to open surgery	—
Davila 2017 [4]	273 ± 39/144 ± 50	—	103 ± 18/97 ± 31 (suspension time)	—	30 ± 7/0	100/82	—	—	0/53 (7/13)	1 ± 1.5/2 ± 1	No transfer to multi‐port or to open surgery	—
Gao 2021 [1]	223.56 ± 15.43/248.61 ± 20.89	—	—	—	—	217.25 ± 16.77/294.74 ± 24.00	—	6.25 (2/32)/5.71 (2/35)	21.88 (7/32)/17.14 (6/35)	7.5 ± 1.55/7.17 ± 1.64	No transfer to multi‐port or to open surgery	—
Kim 2023 [20]	114.71 ± 44.2/128.69 ± 50.49 81.9 ± 45.07/92.10 ± 55.06 134.55 ± 63.39/160.42 ± 61.62	—	—	—	3.66 ± 1.37/0 3.59 ± 2.08/0 3.48 ± 1.98/0	—	1.53 ± 1.04/1.5 ± 1.13 1.59 ± 1.08/1.88 ± 1.03 1.75 ± 1.2/2.85 ± 1.62	—	0/1.4 (2/148) 0/0 0/0	4.54 ± 1.01/4.55 ± 1.26 4.55 ± 1.86/4.37 ± 0.96 4.57 ± 1.12/5.17 ± 2.65	No transfer to multi‐port or to open surgery	—
Woodall 2015 [21]	154/117/128	—	—	—	—	—	—	—	—	—	No transfer to multi‐port or to open surgery	25 509.2/17 584.09/17 226.18
El Hachem 2016 [5]	85.6/78.6 (56.3/67.4, 95.0/73.5, 101.6/88.8)	28.6/26.8 (28.2/24.9, 34.0/28.1, 30.1/27.2)	—	—	—	73.6/104.1 (30.0/86.4, 100.0/114.7, 96.2/107.0)	—	3.03/1.69	6.06/0	(> 24 h) 3.03/0	No transfer to open surgery (The 8 RSS cases that were aborted or converted multi‐port surgery) 24.24/0	10 743/8938 Case–Control Matching (adnexal‐only procedures 18 585/15 450, Benign hysterectomy 21 412/14 623, Malignant hysterectomy without lymph nodes 23 265/16 810, malignant hysterectomy with lymph nodes 23 321/18 385)

### 3.2 Quality Assessment

The 16 studies [1, 4, 5, 6, 10, 11, 12, 13, 14, 15, 16, 17, 18, 19, 20, 21] included all had clear inclusion and exclusion criteria. The NOS (Newcastle‐Ottawa Scale) was used to evaluate the methodological quality. The results showed that among the 15 retrospective cohort studies, 5 had the same score of 9 points, and 2 had the same score of 8 points, both of which were scored in items 1–7. They were the 12th article respectively [4], because the single‐site Da Vinci group was followed up for only 6 months, while the single‐site laparoscopic group was followed up for 12 months. The 14th article [20], because of the limitations of longitudinal follow‐up of each patient. The remaining 8 articles, because no follow‐up was mentioned, all scored 7 points. One retrospective case–control study [5] had a NOS score of 8, and the non‐response rate was not described in detail (Table 4).

**TABLE 4:** Methodological quality evaluation table for included studies.

Author, year	Study population selection				Comparability of cohorts based on design or analysis (score)	Evaluation of results			Quality scoring (score)
	Representativeness of the exposed cohorts (score)	Selection of non‐exposed cohorts (score)	Identification of exposure factors (score)	No outcome events occurred before study initiation (score)		Evaluation of outcome events (score)	Adequacy of follow‐up (score)	Integrity of follow‐up (score)	
Gardella 2023 [10]	1	1	1	1	2	1	0	0	7
Paek 2016 [11]	1	1	1	1	2	1	0	0	7
Lopez 2016 [12]	1	1	1	1	2	1	0	0	7
Akdemir 2015 [13]	1	1	1	1	2	1	1	1	9
Gungor 2017 [14]	1	1	1	1	2	1	1	1	9
Paek 2015 [15]	1	1	1	1	2	1	0	0	7
Noh 2023 [16]	1	1	1	1	2	1	0	0	7
Sun 2021 [17]	1	1	1	1	2	1	1	1	9
Lee 2023 [18]	1	1	1	1	2	1	0	0	7
Kang 2023 [19]	1	1	1	1	2	1	1	1	9
Moon 2018 [6]	1	1	1	1	2	1	1	1	9
Davila 2017 [4]	1	1	1	1	2	1	1	0	8
Gao 2021 [1]	1	1	1	1	2	1	0	0	7
Kim 2023 [20]	1	1	1	1	2	1	1	0	8
Woodall 2015 [21]	1	1	1	1	2	1	0	0	7

Author, year	Study population selection				Comparability of case–control based on design or analysis (score)	Measurement of exposure factors			Quality scoring (score)
	Adequately defined cases (score)	Representativeness of cases (score)	Choice of controls (score)	Definition of contrast (score)		Determination of exposure (score)	The same determination method was used for case and control exposures (score)	Non‐response rate (score)	
El Hachem 2016 [5]	1	1	1	1	2	1	1	0	8

### 3.3 Results of the Meta‐Analysis

#### 3.3.1 Total Operation Time

**FIGURE 2:**
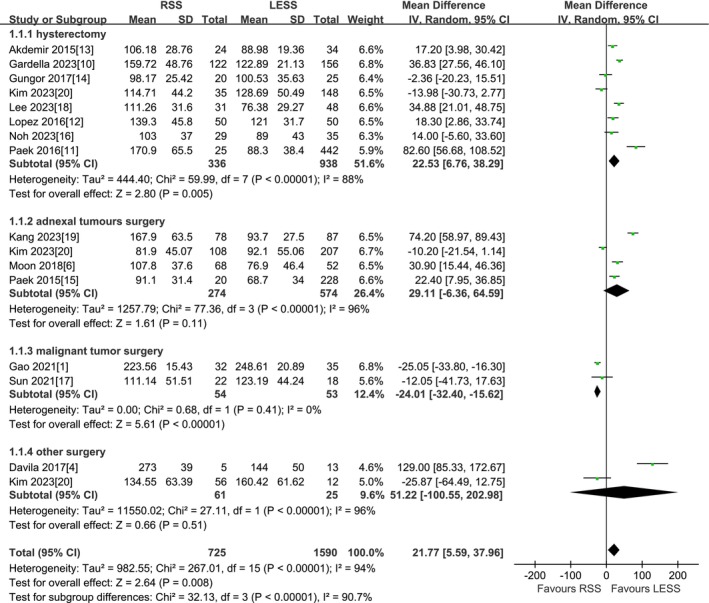
Meta‐analysis of TOT (min). LESS single‐site laparoscopic, RSS single‐site robotic.

A total of 14 studies were included [1, 4, 6, 10, 11, 12, 13, 14, 15, 16, 17, 18, 19, 20]. According to the findings of the present meta‐analysis in a random effects model, the RSS group presented significantly longer total operation time compared to the LESS group (2315 patients MD 21.77 min, 95% CI 5.59–37.96, *p* = 0.008). The results of subgroup analysis by type of surgery indicated that for total hysterectomy, the RSS group presented significantly longer total operation time compared to the LESS group (1274 patients MD 22.53 min, 95% CI 6.76–38.29, *p* = 0.005). For adnexal surgery, neither total operative time was found to be different among the two groups (848 patients MD 29.11 min, 95% CI −6.36 to 64.59, *p* = 0.11). However, for malignant tumor surgery, the RSS group presented significantly shorter total operation time compared to the LESS group (107 patients MD −24.01 min, 95% CI −32.40 to −15.62, *p* < 0.00001). For other surgery, neither total operative time was found to be different among the two groups (86 patients MD 51.22 min, 95% CI −100.55 to 202.98, *p* = 0.51) (Figure 2).

#### 3.3.2 Estimation of Blood Loss

**FIGURE 3:**
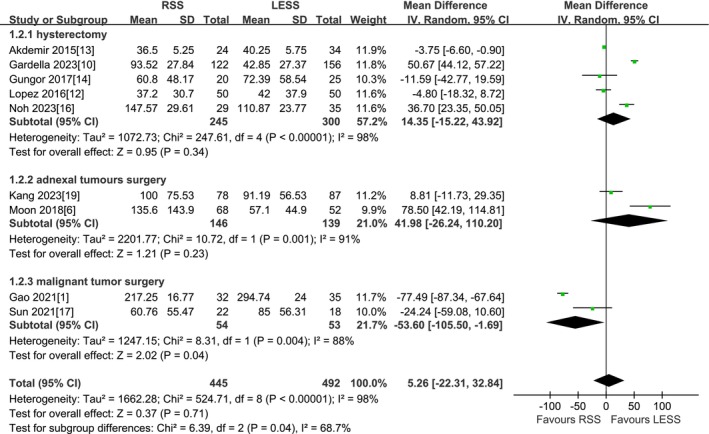
Meta‐analysis of EBL (mL). LESS single‐site laparoscopic, RSS single‐site robotic.

A total of 9 studies were included [1, 6, 10, 12, 13, 14, 16, 17, 19]. According to the findings of the present meta‐analysis in a random effects model, there was no significant difference in estimated blood loss between the RSS group and the LESS group (937 patients MD 5.26 mL, 95% CI −22.31 to 32.84, *p* = 0.71). Subgroup analysis by type of surgery indicated that there was no statistically significant difference between the two groups during total hysterectomy (545 patients MD 14.35 mL, 95% CI −15.22 to 43.92, *p* = 0.34), and during adnexal surgery, there was no statistically significant difference between them (285 patients MD 41.98 mL, 95% CI −26.24 to 110.20, *p* = 0.23). However, during malignant tumor surgery, the RSS group presented significantly lower estimated blood loss compared to the LESS group (107 patients MD −53.60 mL, 95% CI −105.50 to −1.69, *p* = 0.04). The other two studies were excluded because they did not have estimated blood loss, and three studies were excluded because of insufficient data to convert to mean ± standard deviation (Figure 3).

#### 3.3.3 Postoperative Hospital Days

**FIGURE 4:**
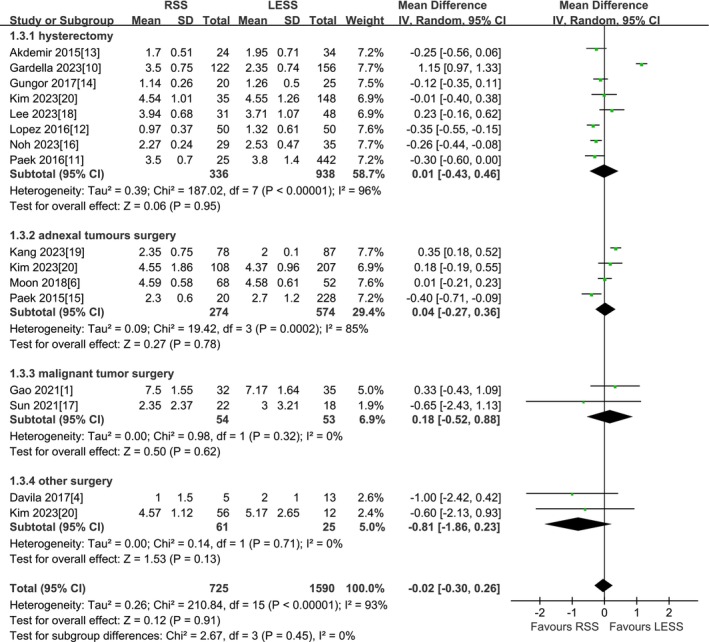
Meta‐analysis of POD (days). LESS single‐site laparoscopic, RSS single‐site robotic.

A total of 14 studies were included [1, 4, 6, 10, 11, 12, 13, 14, 15, 16, 17, 18, 19, 20]. According to the findings of the present meta‐analysis in a random effects model, there was no statistical difference in postoperative hospital days between the RSS group and the LESS group (2315 patients MD −0.02 days, 95% CI −0.30 to 0.26, *p* = 0.91). The results of the subgroup analysis by type of surgery indicated that there was no statistical difference between the RSS group and the LESS group in the subgroups of total hysterectomy, adnexal surgery, malignant tumor surgery, and other surgery (1274 patients MD 0.01 days, 95% CI −0.43 to 0.46, *p* = 0.95) (848 patients MD 0.04 days, 95% CI −0.27 to 0.36, *p* = 0.78) (107 patients MD 0.18 days, 95% CI −0.52 to 0.88, *p* = 0.62) (86 patients MD −0.81 days, 95% CI −1.86 to 0.23, *p* = 0.13) (Figure 4).

#### 3.3.4 Postoperative Complications

**FIGURE 5:**
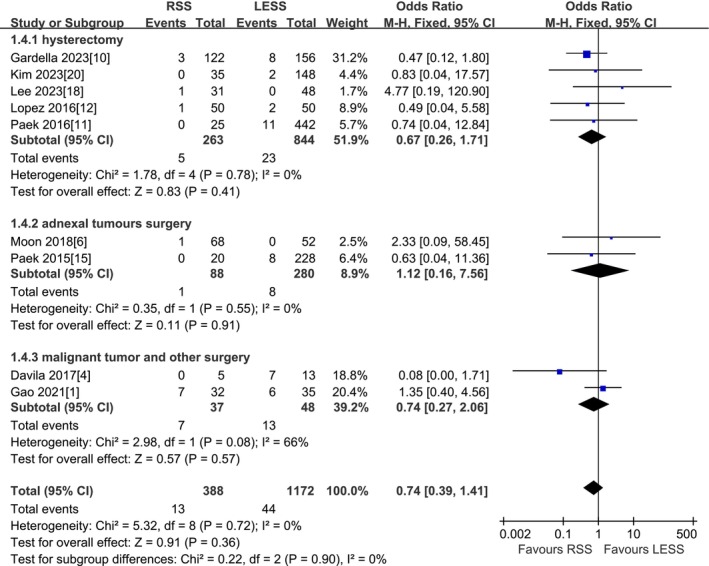
Meta‐analysis of postoperative complications (%). LESS single‐site laparoscopic, RSS single‐site robotic.

A total of 9 studies were included [1, 4, 6, 10, 11, 12, 15, 18, 20]. According to the findings of the present meta‐analysis in a fixed effects model, there was no statistical difference in postoperative complications between the RSS group and the LESS group (1560 patients OR 0.74, 95% CI 0.39–1.41, *p* = 0.36). The results of the subgroup analysis by type of surgery indicated that there was no statistical difference between the RSS group and the LESS group in subgroups undergoing total hysterectomy, adnexal surgery, malignant tumor surgery, and other operations surgery (1107 patients OR 0.67, 95% CI 0.26–1.71, *p* = 0.41) (368 patients OR 1.12, 95% CI 0.16–7.56, *p* = 0.91) (85 patients OR 0.74, 95% CI 0.27–2.06, *p* = 0.57). The other 5 studies were not included due to no postoperative complications (Figure 5).

#### 3.3.5 Mean Serum Hemoglobin Drop

**FIGURE 6:**
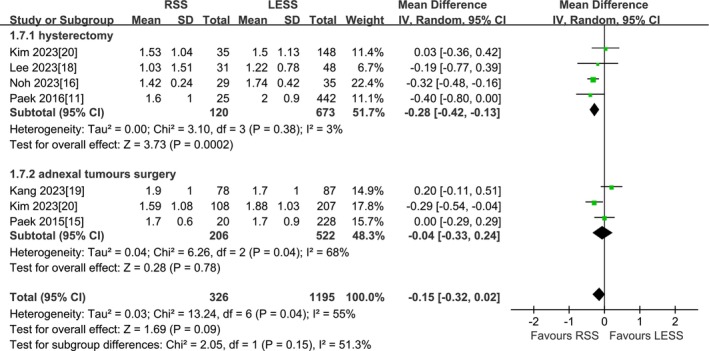
Meta‐analysis of the mean serum hemoglobin drop (g/dL). LESS single‐site laparoscopic, RSS single‐site robotic.

A total of 6 studies were included [11, 15, 16, 18, 19, 20]. According to the findings of the present meta‐analysis in a random effects model, there was no statistical difference in mean serum hemoglobin drop between the RSS group and the LESS group (1521 patients MD −0.15 g/dL, 95% CI −0.32 to 0.02, *p* = 0.09). The results of the subgroup analysis by type of surgery indicated that, in total hysterectomy, the mean serum hemoglobin drop in the RSS group was significantly less than that in the LESS group (793 patients MD −0.28 g/dL, 95% CI −0.42 to −0.13, *p* = 0.0002). However, for adnexal surgery, neither mean serum hemoglobin drop was found to be different between the two groups (728 patients MD −0.04 g/dL, 95% CI −0.33 to 0.24, *p* = 0.78) (Figure 6).

#### 3.3.6 Time for Hysterectomy

**FIGURE 7:**
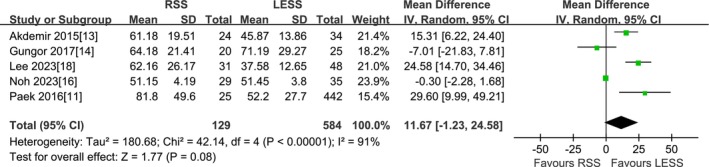
Meta‐analysis of time for hysterectomy (min). LESS single‐site laparoscopic, RSS single‐site robotic.

A total of 5 studies were included [11, 13, 14, 16, 18]. According to the findings of the present meta‐analysis in a random effects model, the time for hysterectomy in the RSS group was slightly longer than that in the LESS group, but the difference was not statistically significant (713 patients MD 11.67 min, 95% CI −1.23 to 24.58, *p* = 0.08) (Figure 7).

#### 3.3.7 Time for Cuff Closure

**FIGURE 8:**
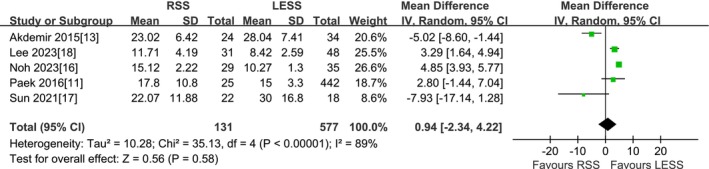
Meta‐analysis of time for cuff closure (min). LESS single‐site laparoscopic, RSS single‐site robotic.

A total of 5 studies were included [11, 13, 16, 17, 18]. According to the findings of the present meta‐analysis in a random effects model, the time for cuff closure in the RSS group was slightly longer than that in the LESS group, but the difference was not statistically significant (708 patients MD 0.94 min, 95% CI −2.34 to 4.22, *p* = 0.58) (Figure 8).

#### 3.3.8 Total Hospital Costs

Only 2 studies were included [5, 21]. And no meta‐analysis was performed because one of them did not describe the data in detail as mean or median [21].

#### 3.3.9 The Other Results

**FIGURE 9:**
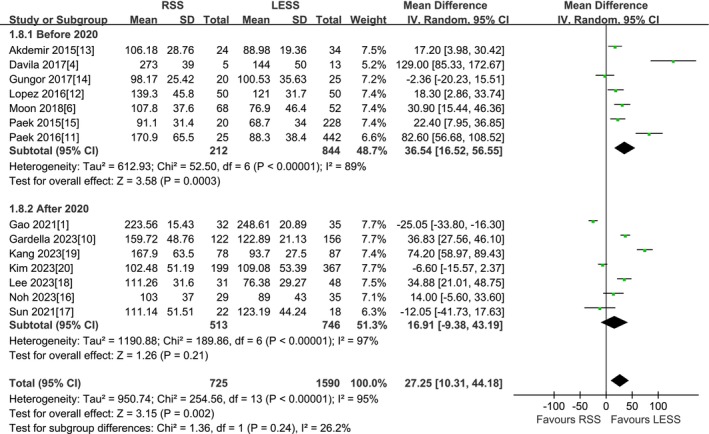
Meta‐analysis of subgroup analysis for TOT based on time of study publication (min). LESS single‐site laparoscopic, RSS single‐site robotic.

**FIGURE 10:**
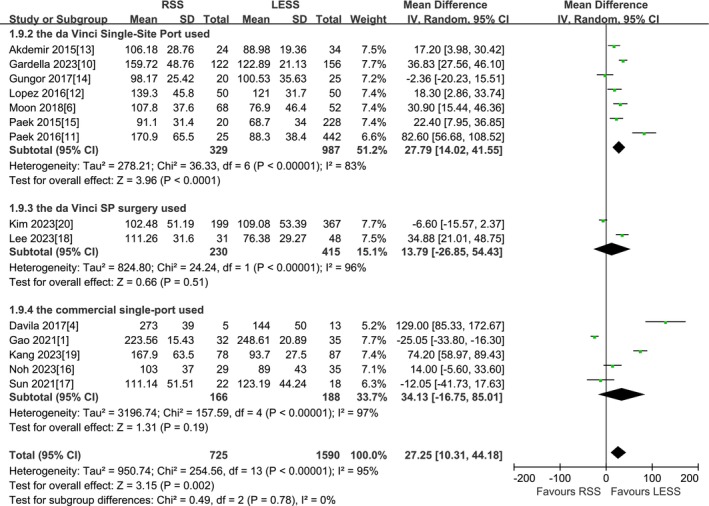
Meta‐analysis of subgroup analysis for TOT based on different single‐site ports (the Da Vinci single‐site port or the commercial single‐site port) and robotic surgical system (SP) (min). LESS single‐site laparoscopic, RSS single‐site robotic.

A total of 14 studies were included [1, 4, 6, 10, 11, 12, 13, 14, 15, 16, 17, 18, 19, 20]. Subgroup analysis of total operation time was carried out according to the time of study publication, and the means and standard deviations of the three different types of surgery in the Kim et al. [20] study were combined. According to the findings of the present meta‐analysis in a random effects model, the RSS group presented significantly longer total operation time compared to the LESS group before 2020 (1056 patients MD 36.54 min, 95% CI 16.52–56.55, *p* = 0.0003), while total operative time was not found to be different among the two groups after 2020 (1259 patients MD 16.91 min, 95% CI −9.38 to 43.19, *p* = 0.21) (Figure 9). However, subgroup analysis of total operation time was carried out according to different single point ports and robotic surgical systems, and the means and standard deviations of the three different types of surgery in the Kim et al. [20] study were combined. According to the findings of the present meta‐analysis in a random effects model, for using the traditional Da Vinci special single point port, the RSS group presented significantly longer total operation time compared to the LESS group (1316 patients MD 27.79 min, 95% CI 14.02–41.55, *p* < 0.0001), while for using commercial single point ports other than the Da Vinci dedicated single point port, along with using the fourth‐generation Da Vinci Single‐Port (SP) surgical system, neither showed statistical significance in total operative time between the two groups (354 patients MD 34.13 min, 95% CI −16.75 to 85.01, *p* = 0.19 and 645 patients MD 13.79 min, 95% CI −26.85 to 54.43, *p* = 0.51, respectively) (Figure 10).

## 4 Discussion

The single‐site robotic surgery platform represents today an impressive and evolving technology. The field of ultra‐minimally invasive surgery aims to reduce the surgical and aesthetic impact while maintaining the same procedural complexity. The reduction of invasiveness is one of the main factors to improve the quality of life of patients [22].

And compared with single‐site laparoscopic surgery, single‐site Da Vinci robotic surgery solves problems related to space and motion by providing better visualization and depth perception [23], so it can improve the technical and surgical difficulties caused by single‐site laparoscopic surgery due to limited instrument space and range of motion and other reasons [1]. In addition, Paek et al. [24] study on the learning curve of single‐site laparoscopic surgery showed that surgeons could proficiently master it after 40 cases in single‐site laparoscopic surgery, while the study on the robotic single‐site platform showed that surgeons could reach a high level of competence after a learning curve phase of about 10–15 cases [25]. However, the results of a number of studies and meta‐analysis at the present stage show that there is no unified conclusion on the effect of single‐site Da Vinci robotic surgery compared with single‐site laparoscopic surgery. Therefore, this meta‐analysis conducted a comprehensive analysis of these studies to provide evidence‐based medical evidence for the cost and effectiveness of single‐hole Da Vinci robotic surgery and single‐hole laparoscopic surgery in gynecological diseases.

Our study shows that, compared with the LESS group, the RSS group had a longer total operative time, with a statistically significant difference. However, there were no statistically significant differences between the two groups in terms of estimated blood loss, postoperative hospital days, postoperative complications, and mean serum hemoglobin drop. Subgroup analysis by type of surgery revealed that in total hysterectomy, the RSS group had a longer total operative time than the LESS group, but the mean serum hemoglobin drop was lower in the RSS group, with statistically significant differences observed. However, there were no statistically significant differences between the two groups in terms of estimated blood loss, postoperative hospital days, postoperative complications, time for hysterectomy, and time for cuff closure. In adnexal surgery, there were no statistically significant differences between the RSS and LESS groups in terms of total operative time, estimated blood loss, postoperative hospital days, postoperative complications, and mean serum hemoglobin drop. However, in malignant tumor surgery, the total operation time of the RSS group was significantly shorter than that of the LESS group, and the estimated blood loss of the RSS group was also significantly less than that of the LESS group. Nevertheless, there were no statistically significant differences in postoperative hospital days and postoperative complications. Total operative time, estimated blood loss, postoperative hospital days, and postoperative complications are all important indicators for assessing the effectiveness of surgery, and in total hysterectomy, the total operative time includes setup time, hysterectomy time, vaginal cuff closure time, and docking time, which is why it is considered the primary outcome measure in this study. Therefore, in malignant tumor surgery, RSS is more advantageous than LESS, while there is no evidence to prove that RSS has advantages in total hysterectomy, adnexal surgery, and other surgeries. But, regarding hysterectomy time, this depends on the actual time taken to remove the uterus from the body. Additionally, vaginal cuff closure time is considered the most challenging part of the surgery in terms of intracorporeal suturing techniques [2]. In some of the included studies, this was performed via a vaginal approach, which may have introduced a high degree of deviation. Furthermore, differences in Da Vinci robotic surgical platforms, single‐site equipment, patient selection criteria, institutional experience, and the operational level and proficiency of surgeons led to relatively high heterogeneity in the combined analysis of these studies (*I*_2_ > 50%), indicating significant differences among the studies. This might have affected the results.

In terms of total hospital costs, only two studies were included [5, 21], both of which indicated that the RSS group had higher total hospital costs than the conventional laparoscopic (CL) surgery group (US $25 509.2 vs. 17 226.18) (US $10 743 vs. 8938). Among these studies, the Woodall et al. [21] study indicated that the RSS group had higher total hospital costs than the LESS group (US $25 509.2 vs. 17 584.09). The El Hachem et al. [5] study suggested that, whether for adnexal surgery (US $18 585 vs. 15 450), benign total hysterectomy (US $21 412 vs. 14 623), malignant total hysterectomy (US $23 265 vs. 16 810), or malignant total hysterectomy with pelvic lymph node dissection (US $23 321 vs. 18 385), the total hospital costs were higher in the RSS group than in the CL group. The study also stated that the cost of RSS was determined by the use of surgical instruments, operation time, one‐time consumables cost, and the amortized capital cost per case of the Da Vinci Surgical System. Total operative time and total hospitalization costs were consistently higher for RSS, but reached statistical significance only for benign total hysterectomy and adnexal surgery [5]. Therefore, the results indicated that the RSS is more advantageous than LESS in malignant tumor surgery.

Since five studies included in this meta‐analysis used commercial single point ports other than Da Vinci dedicated single point ports, two studies used the fourth generation Da Vinci SP surgical system, and 85% (6/7) of the studies were published after 2020, the subgroup analysis results of total operation time based on the publication time of the studies suggest that before 2020, compared with the LESS group, the total operation time of the RSS group was presented statistically significantly longer. While after 2020, neither total operative time was found different among the two groups. The subgroup analysis of total operation time based on the difference of the single point ports and robotic surgical systems suggested that the total operation time of the RSS group using the traditional single point port for Da Vinci was presented statistically significantly longer than that of the LESS group, but there was no statistically significant difference in the total operation time between the RSS group using commercial single point ports other than the Da Vinci special single point port, or using the fourth‐generation Da Vinci SP surgical system and the LESS group. Therefore, after 2020, in order to improve the operational efficiency of RSS and LESS surgery, many durable, flexible, and well‐designed commercial single point ports were produced, such as the LAGIS single‐site Port [1, 20] (LAGIS Enterprise Co. Ltd., Taiwan), Glove Port [16, 19] (Nelis, Seoul, Republic of Korea), Uniport [18, 20] (UP04FSP‐A; Dalim, Seoul, Korea), Gel‐POINT mini [4], and so forth, combined with the Da Vinci robotic surgery system, it can be closely guided to the operative target site under stereoscopic laparoscopic magnization, maintain the original plane, and complete the operation [1], and the fourth‐generation Da Vinci SP surgical system has also been approved for marketing. The Da Vinci SP Surgical System is a new system in which a camera and three robotic forceps are inserted into the body through a single small incision to perform surgical manipulation [26]. It is the latest addition to the Da Vinci family, following the S system launched in 2006, the Si system in 2009, and the Xi system in 2014. This system is specifically designed for single‐site surgery. The model eliminates interference between the mechanical arms, integrates advanced imaging technologies such as fluorescence imaging to better visualize tissues and blood flow, and combines the benefits of robotic precision with the advantages of minimally invasive surgery [27]. Compared with the Da Vinci S/Si/Xi systems of Intuitive Surgical, it is capable of performing surgical operations in narrow spaces, such as local anal surgery and head and neck surgeries involving the mouth, ears, nose, and throat [28]. Now it has begun to show its surgical advantages; compared with the LESS group, although the total operation time in the RSS group was not significantly shortened, the difference was not statistically significant compared with that before 2020. Of course, there are also studies on mixed RSS surgery [16], which innovatively change the surgical steps to maximize the advantages of robotic surgery and avoid disadvantages. Kang et al. [19] study showed that RSS surgery may be superior to LESS surgery in terms of preserving ovarian function, especially for patients with mild endometriosis and those undergoing non‐complex surgery. Therefore, with the continuous update of equipment and instruments, the continuous simplification and proficiency of surgical steps, and the approval of the fourth‐generation Da Vinci SP surgical system, single‐site Da Vinci robotic surgery will replace single‐site laparoscopic surgery in gynecology, and it has been shown after 2020.

There are some limitations in this systematic review: ① In the included study, 43% (7/16) were conducted in South Korea, which may introduce publication bias. ② Among them, the third and ninth studies, as well as the eighth and 13th studies, were of different research types from the same research team. Additionally, all 16 studies were retrospective and non‐randomized, which may introduce selection bias. ③ Due to differences in inclusion and exclusion criteria, surgical indications, surgical methods, cuff closure methods, and the use of different Da Vinci robotic surgical platforms (S, Si, Xi, SP), instruments and equipment et al. used in different studies, there may be certain clinical heterogeneity. ④ The surgical results are greatly affected by subjective factors such as the surgeon's skill and experience, etc., and other biases are inevitable. ⑤ Due to limitations in the original data, follow‐up was insufficient in some studies. Additionally, all included studies were in English, which may introduce biases such as language bias, database bias, citation bias, multiple publication bias, and bias in data reporting. Therefore, in order to obtain more comprehensive evidence, it is still necessary to expand the sample, conduct long‐term follow‐up, constantly update and supplement with new studies, and re‐evaluate the systematic review [29].

## 5 Conclusion

To sum up, according to the results of the meta‐analysis of 16 included studies, RSS only shows advantages over LESS in malignant tumor surgery. These advantages include shorter total operation time, less estimated blood loss, and non‐statistically significant differences in total hospitalization cost compared to LESS. However, after 2000, with the constant updates in equipment and instruments and the widespread use of the fourth generation Da Vinci SP surgical system, surgical procedures have become more simplified and refined. As a result, the difference between RSS and LESS in total operation time is no longer statistically significant. This suggests that the era of single‐site Robotic surgery may have arrived after 2020. Due to the limited quality and number of the included studies and methodological differences, the above results should be interpreted with caution. Further large‐scale studies or randomized controlled trials are needed for further evaluation.

## Author Contributions

**Jian‐Zhao Yin:** conceptualization (lead), literature search and screening (equal), data collection (equal), quality assessment (equal), formal analysis (lead), writing – original draft (lead). **Wei‐Feng Gao:** literature search and screening (equal), data collection (equal), quality assessment (equal), formal analysis (support), writing – original draft (support).

## Conflicts of Interest

The authors declare no conflicts of interest.

## Data Availability

The data that support the findings of this study are available from the corresponding author upon reasonable request.
